# Evaluating translational science knowledge gains following an online short course for a general scientific audience

**DOI:** 10.1017/cts.2024.585

**Published:** 2025-01-21

**Authors:** Amanda L. Vogel, Shadab F. Hussain, Jessica M. Faupel-Badger

**Affiliations:** 1 Education Branch, Office of Policy Communications and Education, National Center for Advancing Translational Sciences, National Institutes of Health, Bethesda, MD, USA; 2 Center to Reduce Cancer Health Disparities, National Cancer Institute, National Institutes of Health, Bethesda, MD, USA; 3 Division of Cancer Prevention, National Cancer Institute, National Institutes of Health, Bethesda, MD, USA

**Keywords:** Translational science, translational research, education, training, online education, evaluation, workforce

## Abstract

**Purpose:**

The translational science workforce requires preparation in both core skills for biomedical research and competencies for advancing progress along the translational pipeline. Delivering this content in a highly accessible manner will help expand and diversify the workforce.

**Methods:**

The NCATS Education Branch offers online case study-based courses in translational science for a general scientific audience. The branch updated its course in preclinical translational science with additional content aligned with the NCATS Translational Science Principles, which characterize effective approaches to advance translation. The updated course was offered in 2021 and 2022. The branch also revised the course evaluation to capture knowledge change aligned with the NCATS Translational Science Principles.

**Results:**

Of 106 students, 88 completed baseline or endpoint surveys, with 48 completing both. Most found the online format (*n* = 48; 91%) and case study approach (*n* = 48; 91%) effective. There was a statistically significant increase in knowledge related to the Translational Science Principles (*p < 0.001*). Survey items with the highest endpoint scores reflected the principles on creativity and innovation, efficiency, cross-disciplinary team science, and boundary-crossing collaborations. Findings highlighted the effectiveness of pairing a case study with lectures that offer generalizable strategies aligned with the translational science principles. Students reported the course helped them learn about the trajectory of a drug discovery and development initiative, where their own work fit in, and scientific and operational approaches to apply in their own work.

**Conclusions:**

This online case study-based course was effective in teaching generalizable principles for translational science to students with varied scientific backgrounds.

## Introduction

Translational science is the field that generates innovations that overcome longstanding challenges along the translational research pipeline and in so doing accelerates the process of transforming biomedical research discoveries into health solutions [1,2]. These innovations address scientific, operational, financial, and administrative roadblocks that are common to translational research across diseases and conditions. As a whole, the field aims to transform the way that translational research is done to produce more health solutions more quickly [1,3].

To provide leadership for this work, the translational workforce of the future will need preparation in both core skills for biomedical research (e.g., content expertise, methodological rigor) and competencies for enhancing the translational process [2,4,5]. These include, for example, competencies for designing and implementing innovative research approaches that increase the speed and impact of translational research, leveraging interdisciplinary teams and cross-sectoral partnerships to advance from biomedical research findings to treatments and cures, and ensuring that the products of translational research are relevant to the full diversity of the population and reaching those with greatest need [6–12].

Currently, most National Institutes of Health (NIH)-funded education and training in the translational process occurs in predoctoral, postdoctoral, and early-career scientist training programs. The National Center for Advancing Translational Sciences (NCATS) of the NIH supports these opportunities through a range of extramural awards, including its flagship Clinical and Translational Science Award (CTSA) Program [7,8,13–16]. Translational science education and training opportunities found in these and other leading institutions offer a range of opportunities conveying key competencies and skills in translational science [5].

However, these essential training opportunities have limits to the number and type of participants they can serve, as they require that participants are on-site at specific institutions for long-term commitments at particular training and career stages, and the number of participants is limited by available funding. To rapidly expand and diversify the translational workforce, there is a need to complement these training opportunities with a variety of approaches that expand access to translational science content to a broader range of individuals [2].

Toward addressing this need, the NCATS Education Branch has developed two online short courses in translational science that are open to the broad biomedical research community [17]. Both courses use the case study teaching method to convey core concepts in translational science to a broad scientific audience. This audience includes learners from across training and career stages, from post-baccalaureate fellows through mid-career scientists, with varied disciplinary and professional backgrounds relevant to advancing translation, and with the potential to serve in a range of roles in the translational enterprise (e.g., translational research, science administration, community and patient partnerships) (Table 3). One of these two courses is centered around a case study of preclinical translational science in drug discovery and development, while the other course includes multiple case studies that reflect preclinical, clinical, and population level research. Both courses use a self-paced asynchronous approach, toward maximizing accessibility.

We previously reported on the design and evaluation of the first of these two courses, Principles of Preclinical Translational Science [18,19]. In this paper, we report on enhancements to the course and its evaluation that align with the NCATS Translational Science Principles.

Principles of Preclinical Translational Science teaches key approaches to advancing translation in preclinical drug discovery and development. The case study at the heart of the course is the story of a highly effective preclinical drug discovery and development project led by NCATS and conducted in collaboration with colleagues at multiple academic institutions and the National Cancer Institute. The project aims to develop a first-in-class drug to treat cancer metastasis. The course follows the research initiative from the initial phenotypic observation through phase 1 clinical trials, covering the full trajectory of preclinical drug discovery and development. Faculty include the scientists who conducted the research, who describe the contributions of each participating discipline and the interplay among them; scientific leaders and administrators whose work facilitated the science; and experts in key approaches used to advance the research, such as approaches for effective team-based science and cross-agency collaborations. Together, these faculty convey strategies for success that are applicable to advancing preclinical translational research initiatives more broadly.

The course also includes a rigorous evaluation that assesses students’ satisfaction with the online format and case study teaching method, knowledge change, and self-report impact of participation on scientific skills, knowledge, activities, and career goals [18,19]. More information about the course’s educational goals and approach, the case study at the heart of the course, course design and implementation, faculty characteristics, evaluation approach, and lessons learned for developing case study-based courses in translational science across the translational continuum are provided in prior manuscripts [18,19].

Since the course was first designed, NCATS has developed a set of Translational Science Principles that characterize effective approaches for overcoming scientific and operational challenges that commonly occur across the translational continuum, from T0-T4 (Table 1) [20]. The principles are informed by the approaches that NCATS uses in its scientific activities and that are pursued by our awardees. Elsewhere, we have recommended leveraging the NCATS Translational Science Principles as a framework for core content in translational science education [2]. Taking our own recommendation to heart, we revised our course to ensure that it systematically conveys strategies and approaches aligned with all of the NCATS Translational Science Principles. This involved adding lectures and readings aligned with the principles. We also updated our course evaluation instruments with the addition of a scale that measured students’ knowledge change aligned with the NCATS Translational Science Principles from pre-course to post-course. Here, we describe the enhancements, share the new evaluation scale, and report on the impact of the revised course on students’ knowledge acquisition aligned with the NCATS Translational Science Principles.

**Table 1.** NCATS Translational Science Principles*

**1. Prioritize Initiatives that Address Unmet Needs**	**Focus on pursuing scientific goals that address unmet scientific, patient or population health needs.** Example approaches: Scientific Needs: Contribute to research advances in under-investigated areas of science or on scientific questions that present unique research challenges or disincentives (e.g., currently untreatable diseases; de-risking targets). Patient and Population Health Needs: Advance research to develop solutions for unmet patient and population health needs.
**2. Produce Generalizable Solutions for Common and Persistent Challenges**	**Develop innovations that address persistent challenges to advancing translational progress that are found across multiple research initiatives or projects, or span research on multiple diseases, or conditions.** Example approaches: Across Multiple Projects or Initiatives: Advance research by identifying, developing, and/or testing solutions to common bottlenecks or roadblocks that have stymied multiple projects. These may be scientific, operational, or administrative in nature. Across Diseases or Conditions: Approach research challenges and develop solutions by seeking commonalities across research projects on a range of diseases or conditions. Organizational Environment: Enable development and testing of generalizable solutions through organizational policies, organizational structure, and shared resources.
**3. Emphasize Creativity and Innovation**	**Leverage creativity and innovation in research design, conduct, and facilitating factors, with the goal of increasing the impact of the research.** Example approaches: Research Design and Implementation:Pose innovative research questions and develop and implement innovations in research methods, technologies, and approaches that increase the impact of the research, as through pursuit of paradigm-changing goals, or innovations that are generalizable to advancing research across multiple initiatives, diseases, and conditions. Research Processes and Structures: Develop and implement innovations in research team interactions, leadership and management, partnerships, and operations that facilitate and support the quality and impact of the research. Organizational Environment: Enable creativity and innovation through policies that encourage innovations and do not penalize failures.
**4. Leverage Cross-Disciplinary Team Science**	**Engage team members with expertise across disciplines, fields, and professions to produce research that advances translation along the translational research continuum.** Example approaches: Leverage Broad Expertise: Engage colleagues from across disciplines, fields, and professions to advance research along the translational continuum. This may involve leveraging scientific, administrative, financial, and operational expertise. Integrate Knowledge: Integrate concepts, theories, methods, technologies, and approaches from the range of disciplines, fields, and professions that can contribute to advancing the research goals. Leverage knowledge integration to produce more holistic research designs and findings that are therefore more relevant to real-world applications. Organizational Environment: Enable team science via organizational policies, team leadership and management, shared instrumentation and space, and recognition and reward systems.
**5. Enhance the Efficiency and Speed of Translational Research**	**Implement evidence-informed practices and scientific and operational innovations to accelerate the pace of translational research.** Example approaches: Scientific Efficiencies: Develop and implement innovations in scientific approaches, methods and technologies that accelerate the pace of translational research. Collaboration Efficiencies: Implement evidence-informed practices to enhance the speed at which collaborations and teams form, develop a shared vision and goals, effectively communicate, and coordinate work tasks. Project Management Efficiencies: Implement milestone-based decision making to enable rapid agreement on go/no-go decisions, to enable resources to be used most efficiently. Organizational Environment: Reward efficiency, enable rapid failures, and encourage redirection of resources to subsequent attempts.
**6. Utilize Boundary-Crossing Partnerships**	**Leverage collaborations across agencies and sectors and engage patients and communities in research to advance translational progress.** Example approaches: Cross-Sectoral Partnerships: Form partnerships across government, universities, and industry to leverage varied expertise and resources to accelerate translational progress. Implement evidence-informed practices for effective cross-sectoral partnerships. Patient and Community Engagement: Involve impacted patients and communities as research collaborators to enable research advances across the translational continuum (e.g., via disease registries, clinical trials participation, and intervention design). Implement evidence-informed practices for patient- and community-engaged research. Organizational Environment: Enable and incentivize boundary-crossing partnerships via leadership, policies, and recognition and reward systems.
**7. Use Bold and Rigorous Research Approaches**	**Develop ambitious research questions and address them with rigorous and robust methods toward generating reproducible findings that contribute to advancing translation.** Example approaches: Bold Scientific Approaches: Explore ambitious research goals that have the potential to produce major advances and/or paradigm shifts. These may be in areas of research that have been historically intractable or where there are high risks of failure. Rigor and Reproducibility: Employ rigorous and robust approaches to generate reproducible findings and high-quality FAIR (findable, accessible, and interoperable, reusable) data that will enable the research to advance translational progress regardless of whether the initial research objective is met (e.g., learning from failures). To the maximum extent possible, disseminate all parameters utilized to conduct the research (e.g., materials, subjects), research methods and conditions, authentication of reagents and biological resources, data sets, metadata, analytic approaches, and statistical tools used for experimentation and data interpretation, results and conclusions, to facilitate reproducibility and/or inform future study designs. Organizational Environments: Enable rigorous testing of bold, paradigm-challenging ideas, including high-risk high-reward opportunities. Encourage reporting of information necessary for reproducibility toward informing future studies.
**8. Prioritize Diversity, Equity, Inclusion, and Accessibility (DEIA)**	**Leverage diversity, equity, inclusion, and accessibility to produce research outcomes that are relevant to the full diversity of the population.** Example approaches: Research Priority Setting: Include diverse perspectives in research priority setting, such as through partnerships with diverse collaborators, so research investments represent diverse population and patient health needs. Scientific Approaches: Integrate DEIA into the development of research resources and design and implementation of studies, across the translational research continuum. Workforce and Operations: Advance DEIA in the scientific workforce to leverage maximum expertise. Implement best practices in outreach, recruitment and hiring, development, and retention to sustain a diverse and talented workforce.

* The NCATS Translational Science Principles are a living product that will continue to be refined as our understanding of translational science develops. The first seven principles shown in this table were used as the framework to enhance the course and evaluation instrument. After we implemented these enhancements, we introduced the eighth principle on diversity, equity, inclusion, and accessibility (DEIA), shown in this table. We are currently developing enhancements to the course and evaluation instrument to include this principle, and these will be shared with the scientific community when complete. The most recent version of the NCATS Translational Science Principles is available on the NCATS website [20].

## Methods

### Course modifications

Building upon the course design described in our prior publications [18,19], we enhanced the course focus on the NCATS Translational Science Principles by adding new lectures and readings that provided content on: (1) the NCATS Translational Science Principles as a framework for understanding core approaches to advance progress along the translational pipeline; (2) evidence-informed approaches for fostering creativity and innovation in science teams; (3) the role of interdisciplinary team science to advance translational progress, and related effective practices; and (4) characteristics of the NCATS organizational environment that enable scientists to implement approaches aligned with the Translational Science Principles. This complemented existing content and ensured that the course addressed all seven Translational Science Principles that existed at that time. Overall, enhancements built out two tracks of lectures in the course. One track conveyed the case study and was delivered by scientists who participated in the research described in the case study. The other track shone a spotlight on the NCATS Translational Science Principles reflected in the case. These lectures conveyed generalizable approaches to implement the NCATS Translational Science Principles in practice and were delivered by experts with backgrounds in business and management sciences, team science, and legal aspects of cross-sectoral collaboration in drug discovery and development. Additional details about the course are provided in prior publications and on the NCATS Education Branch webpages. This revised course was offered in summer 2021, fall 2021, and spring 2022, and evaluation data from these sessions are included in this publication.

### New pilot scale to assess translational science knowledge change

Enhancements were made to the preexisting course evaluation instruments to reflect the course’s stronger focus on the NCATS Translational Science Principles [18,19]. In addition, we refined survey questions related to student characteristics and degree of course participation. These enhancements were made while retaining the overall evaluation approach we previously reported, informed by the Kirkpatrick Evaluation Model. The model identifies four levels of outcomes and impacts for educational offerings: (1) satisfaction with the course; (2) knowledge acquisition; (3) behavioral and attitudinal change; and (4) impact on performance [21].

Data collection comprised baseline and endpoint self-administered online student surveys disseminated in the first and last weeks of the course. Students who did not complete the baseline survey before starting the course were counted as non-responders. Students who did not complete the endpoint survey within one month of completing the course were counted as non-responders. Survey instruments collected both quantitative and qualitative data on student characteristics, learning goals, degree of participation, satisfaction with the course, knowledge change, and impacts of participation on the approaches participants planned to use in their current and future scientific work, as well as their longer-term research and professional goals.

To reflect the stronger course emphasis on the NCATS Translational Science Principles, we added a newly constructed pilot scale to both the baseline and endpoint survey instruments, called the Translational Science (TS) Knowledge Scale (Table 2). This scale was included along with a preexisting scale that assessed knowledge of the science described in the course case study, such as knowledge of drug discovery and development and clinical trials, which we called the course case (CC) knowledge scale.

**Table 2.** Pilot Translational Science Knowledge Scale

*Subscale 1: General Concepts in Translational Science*	
1. The stages of research on the translational spectrum, and how they interact	
2. The difference between “translational research” and “translational science”	
3. The definition of “**scientific** principles of translational science”	
4. The definition of “**operational** principles of translational science”	
5. Key **scientific** challenges in translational research that translational science aims to address	
6. Key **operational** challenges in translational research that translational science aims to address	

*Subscale 2: Translational Science Approaches, Strategies and Methods*	*Principle(s) Reflected in each Item*
1. Approaches to **advance research in under-investigated areas of science** (e.g., where there are research gaps, disincentives)	Prioritize initiatives that address unmet needs
2. Approaches to develop research initiatives that **address unmet patient or population health needs** (e.g., rare diseases and health disparities)	Prioritize initiatives that address unmet needs
3. Strategies to conduct research that **produce solutions to challenges that have stymied multiple projects** (e.g., roadblocks, bottlenecks, and disincentives)	Produce generalizable solutions for common and persistent challenges
4. Strategies to **increase creativity and innovation** in translational research	Emphasize creativity and innovation
5. Approaches to **increase the efficiency or accelerate the pace** of translational research	Enhance the efficiency and speed of translational research
6. Approaches to **take evidence-informed risks** in scientific goals, research questions, and methods toward advancing translational research	Use bold and rigorous research approaches
7. Approaches to **engage in cross-disciplinary science** that integrate disciplinary expertise to produce research that is more innovative, impactful, and/or accelerates advancement along the translational spectrum	Leverage cross-disciplinary team science
8. Approaches to **compose effective cross-disciplinary and cross-agency science teams** to advance translational research projects	Leverage cross-disciplinary team science; Utilize boundary-crossing partnerships
9. Strategies to **enhance collaborative interactions** among members of translational research teams in ways that help to increase the team’s scientific effectiveness.	Leverage cross-disciplinary team science; Utilize boundary-crossing partnerships
10. Approaches to **form and sustain effective and productive cross-agency partnerships (among government agencies, universities, and/or industry)** to advance translational research	Utilize boundary-crossing partnerships
11. Approaches to **develop and sustain a culture of science** at the level of an initiative, organization, or agency that enables scientists to engage in the strategies and approaches listed in this table toward advancing translational research.	Relevant to application of each principle

The pilot TS Knowledge Scale was derived from the NCATS Translational Science Principles. It included two subscales. The first subscale included six items that captured knowledge of general concepts in translational science, such as the fact that translational science is not synonymous with translational research, and that translational science includes both scientific and operational approaches that advance translational progress. The second subscale included 11 items that captured knowledge of specific approaches, strategies, and methods aligned with the seven NCATS Translational Science Principles that existed when the course was updated. Response options were on a five-point Likert Scale, from no knowledge to expert knowledge. There was no reverse scoring. As this was a pilot scale, no reliability or validity testing was conducted, but this testing is planned for the future, with a version of the scale that also reflects the 8^th^ Translational Science Principle on DEIA. The updated baseline and endpoint survey instruments are provided in the Supplementary Materials.

### Hypotheses

The course evaluation tested five hypotheses, as follows:

H1: Overall, there will be a statistically significant increase in scores on the pilot *TS Knowledge Scale, from baseline to endpoint*.

H2: Students with fewer years of experience in translational research will have greater increases in scores on (a) the *TS Knowledge Scale* and (b) the *CC knowledge* scale compared to students with more years of experience in translational research.

H3: Students with and without a background in cancer biology will have similar increases in scores on (a) the *TS Knowledge Scale* and (b) *the CC knowledge* scale.

H4: Students with no prior background in drug discovery and development will have greater increases in scores on (a) the *TS Knowledge Scale* and (b) *the CC knowledge scale* compared to students with this background.

H5: Students with lower baseline scores on the TS Knowledge Scale will have greater increases in scores on (a) the *TS Knowledge Scale* and (b) the *CC knowledge scale* compared to students with higher baseline scores on the TS Knowledge Scale.

### Data analysis

Quantitative data analyses were conducted in SAS and included examination of frequencies and paired t-tests. The paired t-tests were used to test hypotheses 1–5. Qualitative data analyses comprised thematic analysis of text responses to open-ended questions about the impacts of the course, with a focus on identifying themes related to the NCATS Translational Science Principles. Excel was used to support these analyses. This educational research received an exemption from the NIH Institutional Review Board (project number P205038).

## Results

### Sampling frame and sample

A total of 106 students participated in the three course sessions. Of the 88 students who responded to at least one of the surveys, 37 completed the baseline survey only, 2 completed the endpoint survey only, and 48 students completed both surveys. An additional student completed the endpoint survey, meaning 51 students in total completed the endpoint survey. This additional student did not complete the baseline survey but completed most of the items. Thus, the student’s baseline data were included in the descriptive analyses, where provided. The hypothesis testing to assess change in knowledge was conducted only for the 48 students who completed both surveys.

### Respondent characteristics

Respondents reported a variety of academic and career backgrounds, as well as demographic characteristics (Table 3).

**Table 3.** Student Characteristics at Baseline

Training, Disciplinary and Professional Background (*n* = 85)	No. (%)
**Highest degree**	
PhD	39 (46)
Bachelors	26 (31)
Masters	9 (11)
MD	7 (8)
MD/PhD	2 (2)
Other	2 (2)
**Disciplinary training, by highest degree**	
Biology, Microbiology, Molecular Biology	29 (34)
Medicine	11 (13)
Engineering, Biomedical Engineering or Chemical Engineering	8 (9)
Biochemistry	7 (8)
Chemistry	6 (7)
Computer Science or Informatics	3 (4)
Neuroscience	3 (4)
Nursing	2 (2)
Pharmacology or Toxicology	2 (2)
Psychology	2 (2)
Public Health	2 (2)
Other	11 (13)
**Years since highest degree**	
0–5 years	52 (61)
6–10 years	9 (11)
11–15 years	13 (15)
16–20 years	4 (5)
More than 20 years	7 (8)
**Work sector**	
Academia	43 (51)
Government	28 (33)
Industry/business/private sector	9 (10)
Non-profit/NGO	4 (5)
Other	1 (1)
**Current student**	
Yes	13 (15)

Background Related to Translational Science (*n* = 86)	No. (%)
**Translational research (TR) experience**	
Less than 1 year	46 (53)
1–2 years	14 (16)
3–5 years	13 (15)
6–10 years	5 (6)
More than 10 years	8 (9)
**Current work contributes to TR**	
Yes	58 (67)
**Currently teach a course in skills for TR**	
Yes	3 (3)
Background Related to the Course Case Study (*n* = 86)	No. (%)
**Background in Cancer Biology**	
I have been involved in conducting cancer biology research	34 (39)
I have been involved in conducting other cancer research	21 (24)
I have academic training in cancer biology	17 (20)
I have been involved in providing cancer patient care	6 (7)
None of the above	35 (40)
**Background in Drug Discovery and Development (D&D)**	
I have been involved in conducting drug D&D research	34 (39)
I have academic training in drug D&D	21 (24)
I have been involved in business, administrative, or legal work around drug D&D	12 (14)
None of the above	36 (41)
Demographic Characteristics (*n* = 85)	No. (%)
**Race/Ethnicity (US residents/citizens only) – respondents were asked to check all that apply**	
White	42 (49)
Asian	24 (28)
Black or African American	4 (5)
Hispanic or Latino	4 (5)
Other	1 (1)
Prefer not to say	4 (5)
Does not apply – not a US resident or citizen	6 (7)
**Working/studying abroad**	
Yes	7 (8)
**Gender identity**	
Man	22 (26)
Woman	60 (71)
Non-binary or prefer not to say	3 (3)

### Respondents’ learning goals, engagement with the course, and course satisfaction

Respondents had multiple learning goals for taking the course (Table 4). Degree of participation in each component of the course varied with whether the component was required (i.e., lectures and required readings) or optional (i.e., recommended readings and submitting questions for Live Q&A sessions with course faculty on Zoom). To assess engagement with course content, students were asked how frequently they discussed concepts or content learned in the course outside of class. About a third (35%) reported that they discussed course concepts outside of class during 3 or more of the 7 weeks of the course.

**Table 4.** Learning goals, degree of participation, and course satisfaction

Baseline evaluation questions (*n* = 86)	No. (%)
**Learning goals**	
Obtain knowledge and skills that I can apply in my future career	57 (66)
Obtain knowledge and skills that I can apply in my current work	52 (60)
Get an introduction to translational science	56 (64)
Get an introduction to drug discovery and development	45 (52)
Learn how others are teaching translational research skills, to help me develop/enhance my own course on this topic	16 (18)
Other	1 (1)

Endpoint evaluation questions (*n* = 51)	No. (%)
**Degree of participation in the course**	
Listened to 50%–100% of lectures	46 (87)
Completed 50%–100% of required readings	28 (52)
Completed 50%–100% of recommended readings	11 (20)
Submitted questions for our Live Q&A sessions with faculty on Zoom, 50%–100% of the time	4 (7)
Participated in or listened to one Live Q&A session with faculty	15 (29)
Participated in or listened to both Live Q&A sessions with faculty	15 (29)
**Degree of engagement with course material**	
Did not discuss concepts or content learned in the course outside of class	6 (11)
Discussed concepts or content learned in the course outside of class a few times (1–2 weeks of the course)	28 (53)
Discussed concepts or content learned in the course outside of class some of the time (3 weeks of the course)	10 (19)
Discussed concepts or content learned in the course outside of class many times (4–5 weeks of the course)	6 (11)
Discussed concepts or content learned in the course outside of class most to all of the time (6–7 weeks of the course)	3 (5)
**Online format effectiveness**	
Not at all effective	1 (1)
Somewhat effective	4 (8)
Moderately effective	27 (51)
Very effective	21 (40)
**Case study approach effectiveness**	
Not at all effective	1 (1)
Somewhat effective	4 (8)
Moderately effective	12 (23)
Very effective	36 (68)
**Degree to which course achieved its aim to provide a unique window into TS**	
Slightly	1 (1)
Moderately	22 (31)
Completely	48 (68)
**Course influence on goals for future work**	
Not at all	1 (1)
Slightly	12 (24)
Moderately	27 (53)
Completely	11 (22)
**Degree to which course achieved aims**	
Slightly	3 (6)
Moderately	20 (39)
Completely	28 (55)
**Overall value of course to the respondent**	
Slightly valuable	2 (4)
Moderately value	23 (45)
Extremely valuable	26 (51)

Most respondents reported that the online format and case study approach were moderately or very effective to teach the course content, that the course moderately or completely achieved its aim to provide a unique window into the translational science process, and that the course was moderately or extremely valuable to them, overall.

### Respondents’ change in translational science knowledge, by topic

**Figure 1.**
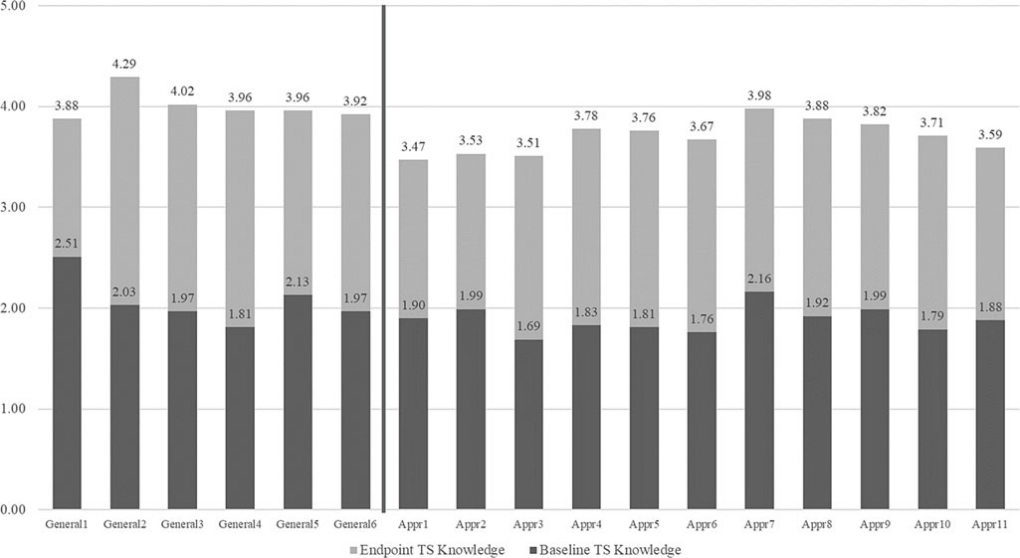
Item means of pilot TS knowledge scale at baseline (*n* = 86) and endpoint (*n* = 51).

Figure 1, below, displays the means at baseline and endpoint for items in each subscale of the pilot TS Knowledge Scale. The items to the left of the vertical bar are in the first subscale, which captured knowledge of general concepts in translational science. The items to the right of the vertical bar are in the second subscale, which captured knowledge of specific approaches, strategies, and methods aligned with the NCATS Translational Science Principles. Overall, the item means are higher at endpoint than at baseline. In addition, items in subscale 1 trended higher at endpoint than items in subscale 2.

On subscale 1, the item with the highest endpoint score reflected self-report knowledge of “the difference between “translational research” and “translational science”” (item 2). This item also had the highest mean difference from baseline to endpoint, showing the greatest increase in knowledge change.

On subscale 2, the item with the highest endpoint score reflected self-report knowledge of “approaches to engage in cross-disciplinary science that integrate disciplinary expertise to produce research that is more innovative, impactful, and/or accelerates advancement along the translational spectrum” (item 7). Additional high-scoring items in subscale 2 at endpoint were, “approaches to compose effective cross-disciplinary and cross-agency science teams to advance translational research projects” (item 8), “strategies to increase creativity and innovation in translational research” (item 4), “approaches to increase the efficiency or accelerate the pace of translational research” (item 5), and “strategies to enhance collaborative interactions among members of translational research teams in ways that help to increase the team’s scientific effectiveness” (item 9). The item with the highest mean difference from baseline to endpoint, showing the greatest increase in knowledge change, was “approaches to compose effective cross-disciplinary and cross-agency science teams to advance translational research projects” (item 8).

### Respondents’ change in scores on the Translational Science Knowledge Scale and course case study knowledge scale (n = 48)

**Figure 2.**
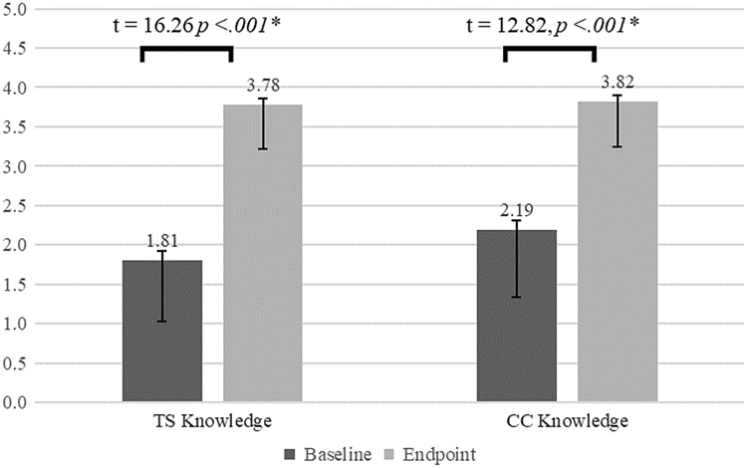
Mean change in students’ translational science knowledge (TS) and course case study related knowledge (CC), results of paired sample T-tests (*n* = 48). Error bars show the standard error of the mean.

Paired t-tests were conducted to test the research hypotheses. Hypothesis 1 posited that there would be a statistically significant increase in scores on the TS Knowledge Scale from baseline to endpoint. Figure 2 displays the results of paired t-tests which confirmed this hypothesis. It also displays a statistically significant increase in scores on the CC knowledge scale from baseline to endpoint.

The remaining hypotheses were tested with paired t-tests comparing pre- and post-course scores on the pilot TS Knowledge Scale and the CC knowledge scale by students’ relevant pre-course knowledge and experience (Table 5). Results revealed that those who had 2 or fewer years of translational research experience had greater increases in TS knowledge (H2a) and CC knowledge (H2b) than those who had 3 or more years of translational research experience. In addition, a background in cancer biology (H3a and H3b) and a background in drug discovery and development (H4a and H4b) had no significant effect on increases in TS knowledge or CC knowledge. These results confirm Hypotheses 3a and 3b, but not Hypotheses 4a and 4b. However, mean differences in scores between those who had no drug discovery background and those who had some drug discovery background were marginally significant for both TS knowledge (*p* = .13) and CC knowledge (*p* = .07). Lastly, those who reported lower baseline TS knowledge had greater increases in both TS knowledge (H5a) and CC knowledge (H5b) than those who reported more TS knowledge at baseline.

**Table 5.** Change in translational science knowledge (TS) and course case study related knowledge (CC) by students’ backgrounds and baseline knowledge (*n* = 48)

	Group 1 (*n*)		Group 2 (*n*)				
Hypotheses	*M*	SD	*M*	SD	*t*-value	CI	*p*-value
	≤2 years of TR experience (*n* = 38)		≥3 years of TR experience (*n* = 10)				
2a. Years of TR experience (TS)	2.14	0.67	1.28	1.07	3.16	[0.64, .96]	.002*
2b. Years of TR experience (CC)	1.80	0.71	0.98	1.16	2.81	[0.68, 1.03]	.007*
	No reported background (*n* = 23)		Some reported background (*n* = 25)				
3a. Cancer biology background (TS)	1.90	0.74	2.02	0.92	-0.50	[0.70,.1.05]	*0.62*
3b. Cancer biology background (CC)	1.64	0.80	1.62	0.95	0.06	[0.74, 1.12]	*0.95*
	No reported background (*n* = 23)		Some reported background (*n* = 25)				
4a. Drug discovery background (TS)	2.15	0.87	1.79	0.77	1.53	[0.69, 1.03]	*0.13*
4B. Drug discovery background (CC)	1.87	0.77	1.41	0.93	1.85	[0.71, 1.07]	*0.07*
	Baseline score: 1–2 (*n* = 32)		Baseline score: 3–5 (*n* = 16)				
5a. Baseline TS knowledge (TS)	2.36	0.61	1.18	0.66	6.15	[0.52, .79]	< .001*
5b. Baseline TS knowledge (CC)	1.96	0.70	0.95	0.81	4.47	[0.62, .93]	< .001*

### Respondents’ written comments about course value and knowledge gains (n = 27)

Of the 51 students who completed the endpoint survey, 27 (53%) provided written comments about the value of the course to them. These 27 students reported a range of training and career stages. About half (*n* = 13) had an MD or PhD, half (*n* = 13) had a bachelor’s degree, and one had a different degree. There were three prevalent themes in their comments, which cut across training and career stages. The first theme was that the course helped students learn about the full trajectory of a translational research initiative in drug discovery and development, and where their own work fit in. For example, one student wrote, “I have primarily worked in a basic setting early in my career and have realized that my interests are rooted closer to the clinic. Using a case study, this course exposed me to the different stages of translation and made me feel a bit more certain that my future is in pre-clinical and clinical trial research.” Another student wrote, “I now know what each group does and what they accomplish, and how to better support projects and help move the process faster.”

The second theme was that the course provided knowledge and skills in scientific and operational approaches to advance translational progress that students could apply to their current or future work. One student wrote, “For someone that plans to work in the pharmaceutical industry, this course was extremely valuable to me for teaching me not only scientific concepts of high-throughput screening, medicinal chemistry, target identification, in vivo/in vitro studies, and clinical trials but also useful concepts about team science, collaboration, leadership, and communication in scientific/professional settings that are applicable to me.” In their comments about the knowledge they gained, students mentioned all seven NCATS Translational Science Principles included in the course. Three of the seven principles were mentioned most often: (1) emphasizing creativity and innovation, (2) leveraging cross-disciplinary team science, and (3) utilizing boundary-crossing partnerships (Table 6). Responses highlighted that the case study teaching method in combination with expert lectures that focused on generalizable approached aligned with the NCATS Translational Science Principles was highly effective to teach the principles.

**Table 6.** Student comments on knowledge gains related to creativity and innovation, cross-disciplinary team science, and boundary crossing partnerships

“I will be using the work on **inducing **creativity** for teams** (active engagement on problem construction leads to creativity).”
“If I’m in future situations where I’m a collaborator or a leader of a team, the concepts that I learned from the course – **implementing team science, fostering creativity, picking good collaborators**, etc. – will influence my decisions and future work.”
“I have been working on a **multidisciplinary** project and participating in this course gave me a new perspective on how the interactions can be developed. I like the **collaborative** nature of the project, but I was always very shy to participate. Now I believe I should be more active because I might have a new perspective that could help the group to advance in the scientific question.”
“Many of the general principles covered later in the course, especially those involving establishing successful **cross-institutional collaborations, and encouraging team science/creativity**, are principles that I can use in my work regardless of the area I choose to work in.”
“It inspired me to see the importance of **creativity and innovation** in science and that with great risk it is expected to have failures too. I think this course gave me a new perspective of the importance of **taking risks in science**.”

The third theme was that the course reinforced or increased students’ interest in a career in translational science. For example, one student wrote, “This course was a great introduction to the field of translational science, and I hope to further my education and perhaps become a translational scientist in the future.” Another wrote, “I confirmed that this is the path I would like to pursue in the future.”

## Discussion

To expand and diversify the translational science workforce, there is a need to enhance access to education and training opportunities in translational science to a broad range of interested individuals. A promising approach is to complement formal training programs in translational science with a range of additional learning opportunities that lower barriers to participation related to location, time, cost, and intensity of participation. These approaches include online education, short courses, workshops, coaching, expert advising, and more.

Audiences for these opportunities include individuals at training and career stages that are earlier or later than those who participate in current formal training opportunities in translational science, individuals with varied disciplinary backgrounds, and those working in a variety of roles in the translational enterprise (e.g., science, administration, community and patient partners). Broadening access to translational science education will benefit the translational enterprise. Approaches that engage individuals earlier in their scientific training create on-ramps into translational science careers, while engaging individuals at mid-career and senior-career stages can bring additional expertise into the field. In addition, involving individuals not only from biomedical research backgrounds but also from backgrounds relevant to science administration and operations (e.g., business and management sciences, organizational engineering) and patient and community engagement has the potential to bring a range of novel expertise into the translational workforce.

Another necessary aspect of enhanced access is to leverage teaching approaches that are effective with varied audiences. The case study teaching method is one such approach. Likewise, the NCATS Translational Science Principles were designed with broad audiences in mind, as they hone complex concepts into a form that is easy to communicate to varied learners. The NCATS Education Branch leverages these approaches to contribute to expanded access to translational science education. While beyond the scope of the course described here, there is also the potential to use the case study teaching method, with its emphasis on storytelling, to effectively engage broader audiences in translational science education and training, such as patient and community partners in research who are essential to advancing the translational enterprise.

**Summary and Interpretation of Findings.** Findings from the course evaluation provide evidence for the effectiveness of the course to convey core concepts in translational science to a broad scientific audience. Findings reflected that the course was most effective in increasing translational science knowledge among students with less translational research experience and lower baseline translational science knowledge. In addition, prior knowledge of cancer biology or the drug discovery and development process did not have a statistically significant impact on students’ increases in TS knowledge and CC knowledge although the case study focused on a cancer drug discovery and development project. Taken together, these findings suggest that the online case study teaching method is effective for teaching translational science core concepts to a general scientific audience. Findings also suggest that students with varied disciplinary backgrounds and areas of expertise can gain TS knowledge from discipline-specific cases when the educational goal is to use these cases to convey generalizable approaches for advancing translation. This suggests that educators can effectively leverage case studies from varied disciplines to convey core principles for translational science, and that the main criteria for case selection are the ability of the case to exemplify key approaches for effective translational science and demonstrate how applying these approaches advances the science, rather than disciplinary match between the case and the course participants.

Looking specifically at change in scores on the pilot TS Knowledge Scale, we found overall increases in translational science knowledge as well as evidence that knowledge increases varied by topic area. In subscale 1, the greatest knowledge gain was around understanding that translational science is related to, but different from, translational research. This is an essential underpinning of the field of translational science, and is a necessary factor in creating the motivation to learn translational science competencies on top of core biomedical research skills.

In subscale 2, which reflected self-report knowledge of specific approaches, strategies, and methods aligned with the Translational Science Principles, the items that scored in the top half at endpoint (5 of 11 items) mapped to four of the seven NCATS Translational Science Principles: creativity and innovation, efficiency and speed, cross-disciplinary team science, and boundary-crossing collaborations. Students’ written comments on their knowledge gains in the course reflected these quantitative findings, with emphasis placed on three out of four of these principles -- all but efficiency and speed. These qualitative data provide some degree of validation for the scale.

### Implications for translational science education and training

There is a longstanding focus on the importance of training the translational science workforce in a range of competencies that extend beyond core training in biomedical research (e.g., literature review, study design, and research ethics) to competencies for advancing translational progress (e.g., generalizable solutions, creativity and innovation, and cross-disciplinary team science) [6,9,22]. Given the ever-growing body of knowledge that is recommended for inclusion in biomedical research graduate training, the question is how to incorporate additional content within temporal limitations.

This course offers a model for how to efficiently convey translational science competencies within a course that also conveys biomedical research knowledge. We found that complementing the case study at the heart of the course with lectures focused on the NCATS Translational Science Principles was highly effective. Top scoring items on the TS Knowledge Scale reflected the content of this second set of lectures, while the case study provided a model of how to implement the principles in practice. The NCATS Education Branch continues to enhance this course as our own understanding of translational science advances. For example, we are currently developing additional course content that will feature DEIA as a critical approach to advance translational goals.

In prior publications, we have recommended that academic institutions create case studies of success from their own settings for teaching purposes [18,19]. While the case study at the center of a course can be adapted to local resources and interests, the focus on conveying generalizable translational science strategies and approaches, and the ability to evaluate related knowledge gains, should remain constant. Biomedical research institutions should leverage the breadth of institutional resources available to them, engaging colleagues from across schools and departments to apply their expertise to deliver content aligned with the Translational Science Principles or other key approaches to advance translational progress. Lectures or seminars that focus purely on conveying generalizable principles can also be provided independent of a course such as the one described here and used to complement experiential learning, highlighting generalizable lessons learned.

There are legitimate concerns about degree of participation and engagement with asynchronous online learning. Predictably, we found that degree of participation in each component of the course varied with whether the component was required or optional. We found a fairly high level of engagement in the course, with about a third of students reporting that they discussed course concepts with individuals who were not classmates anywhere from 3 to 7 weeks out of the 7-week course. Course content varied significantly from week to week, including scientific lectures and lectures on Translational Science Principles. There may have been weeks that generated more interaction outside of the course, or that resonated with particular students. Toward encouraging engagement, students received a weekly email at the start of each week with a summary of course progress and reminders of upcoming assignments. In addition, students who completed all required components of the course received a digital badge through the NCATS Digital Badging Program.

### Implications for further development of the TS knowledge scale and future evaluations

This evaluation piloted the TS Knowledge Scale for the first time. The scale offers the advantage of assessing knowledge change on a wide range of translational science concepts in a single scale. Yet as a pilot scale, there are limitations in interpreting results. To validate the scale, it can be used in evaluations for other courses teaching the NCATS Translational Science Principles to establish content validity, and scale scores can be compared to similar outcome measures to establish concurrent validity.

We are currently working to refine and test the scale. Since this scale was developed, we have added an eighth NCATS Translational Science Principle on DEIA, as shown in Table 1. We have also refined the content of the preexisting seven principles. The scale will be revised to reflect these updates. In addition, the current version of the scale assesses self-report knowledge, rather than objective measures of knowledge. We are modifying the scale to reflect self-efficacy to implement the NCATS Translational Science Principles in practice. We anticipate this will produce more accurate data while still maintaining a structure that is low-burden to respondents and therefore suitable to a short online course. Our next steps are to test and validate the revised scale in our courses at NCATS.

Our goal in sharing the pilot version of the TS Knowledge Scale is to provide a model for evaluating learners’ knowledge gains on a range of generalizable strategies and approaches to advance translational progress. As the NCATS Translational Science Principles were developed with a focus on creating solutions to common challenges in translational research across the translational continuum, the scale should be applicable to a range of translational science education and training opportunities.

The current pilot version may be useful to colleagues as-is or to inform their own work to develop scales reflecting knowledge of generalizable approaches to overcome translational challenges and advance translational progress. We are interested in hearing about the related experiences of colleagues in the translational science education and training community. Overall, translational science education and training will benefit from rigorous evaluation of educational offerings, including further evaluation of the case study-based approach to teach generalizable translational science strategies and approaches. This work will be enriched through evaluation of educational opportunities that leverage cases reflecting science across the translational continuum and that engage varied audiences who all play a role in advancing the translational enterprise.

## Supporting information

Vogel et al. supplementary material Vogel et al. supplementary material

## Supplementary material

The supplementary material for this article can be found at https://doi.org/10.1017/cts.2024.585.

## References

[1] Austin CP. Translating translation. Nat Rev Drug Discov. 2018;17(7):455–456. doi: 10.1038/nrd.2018.27. 29674698 PMC6023744

[2] Faupel-Badger JM , Vogel AL , Austin CP , Rutter JL. Advancing translational science education. Clin Transl Sci. 2022;15(11):2555–2566. doi: 10.1111/cts.13390. 36045637 PMC9652430

[3] Austin CP. Translational misconceptions. Nat Rev Drug Discov. 2021;20(7):489–490. doi: 10.1038/d41573-021-00008-8. 33462425

[4] Tsevat J , Smyth SS. Training the translational workforce: expanding beyond translational research to include translational science. J Clin Transl Sci. 2020;6(4):360–362. doi: 10.1017/cts.2020.31. PMC7681113 33244418

[5] Vogel AL , Haynes BM , Hussain SF , et al. Areas of strength and opportunities for growth in translational science education and training: results of a scoping review from the NCATS education branch. Clin Transl Sci. 2023;2(9):1526–1546. doi: 10.1111/cts.13570. PMC10499424 37533169

[6] Meyers FJ , Begg MD , Fleming M , Merchant C. Strengthening the career development of clinical translational scientist trainees: a consensus statement of the clinical translational science award (CTSA) research education and career development committees. Clin Transl Sci. 2012;5(2):132–137. doi: 10.1111/j.1752-8062.2011.00392.x. 22507118 PMC3771692

[7] Sancheznieto F , Sorkness CA , Attia J , et al. Clinical and translational science award T32/TL1 training programs: program goals and mentorship practices. J Clin Transl Sci. 2022;6(1):e13. doi: 10.1017/cts.2021.884. 35211339 PMC8826009

[8] Sorkness CA , Scholl L , Fair AM , Umans JG. KL2 mentored career development programs at clinical and translational science award hubs: practices and outcomes. J Clin Transl Sci. 2020;4(1):43–52. doi: 10.1017/cts.2019.424. 32257410 PMC7103475

[9] CTSA ECCWG. Core Competencies for Clinical and Translational Research. https://clic-ctsa.org/education/competencies. Accessed May 20, 2022.

[10] Pusek S , Knudson B , Tsevat J , et al. Personalized training pathways for translational science trainees: building on a framework of knowledge, skills, and abilities across the translational science spectrum. J Clin Transl Sci. 2020;4(2):102–107. doi: 10.1017/cts.2019.445. 32313699 PMC7159805

[11] Begg MD , Bennett LM , Cicutto L , et al. Graduate education for the future: new models and methods for the clinical and translational workforce. Clin Transl Sci. 2015;8(6):787–792. doi: 10.1111/cts. 26643714 PMC4709034

[12] Begg MD , Crumley G , Fair AM , et al. Approaches to preparing young scholars for careers in interdisciplinary team science. J Investig Med Jan. 2014;62(1):14–25. doi: 10.2310/JIM.0000000000000021. PMC3970261 24169319

[13] NCATS. PAR-21-339. Limited Competition: NCATS Clinical and Translational Science Award (CTSA) Program Research Education Grants Programs (R25 - Clinical Trial Not Allowed). https://grants.nih.gov/grants/guide/pa-files/PAR-21-339.html. Accessed May 20, 2022.

[14] NCATS. PAR-21-336. Limited Competition: Mentored Research Career Development Program Award in Clinical and Translational Science Awards (CTSA) Program (K12 Clinical Trial Optional). https://grants.nih.gov/grants/guide/pa-files/PAR-21-336.html. Accessed May 20, 2022.

[15] NCATS. PAR-21-338. Limited Competition: Ruth L. Kirschstein National Research Service Award (NRSA) Postdoctoral Research Training Grant for the Clinical and Translational Science Awards (CTSA) Program (T32 Clinical Trial Not Allowed). https://grants.nih.gov/grants/guide/pa-files/PAR-21-338.html. Accessed May 20, 2022.

[16] NCATS. PAR-21-337. Limited Competition: Ruth L. Kirschstein National Research Service Award (NRSA) Predoctoral Research Training Grant for the Clinical and Translational Science Awards (CTSA) Program (T32 Clinical Trial Not Allowed). https://grants.nih.gov/grants/guide/pa-files/PAR-21-337.html. Accessed May 20, 2022.

[17] NCATS. Translational Science Training and Education Resources, https://ncats.nih.gov/training-education/resources. Accessed October 6, 2023.

[18] Faupel-Badger JM , Vogel AL , Hussain SF , et al. Teaching principles of translational science to a broad scientific audience using a case study approach: a pilot course from the national center for advancing translational sciences. J Clin Transl Sci. 2022;6(1):1–26. doi: 10.1017/cts.2022.374. PMC9201875 35754433

[19] Vogel AL , Hussain SF , Faupel-Badger JM. Evaluation of an online case study-based course in translational science for a broad scientific audience: impacts on students’ knowledge, attitudes, planned scientific activities, and career goals. J Clin Transl Sci. 2022;6(1):e82. doi: 10.1017/cts.2022.415. 35949657 PMC9305082

[20] NCATS. Translational Science Principles. https://ncats.nih.gov/training-education/translational-science-principles. Accessed May 20, 2022.

[21] Kirkpatrick DL , Kirkpatrick JD . Evaluating Training Programs: The Four Levels. 3rd ed. Oakland, CA: Berrett-Koehler Publishers, 2006.

[22] Gilliland CT , White J , Gee B , et al. The fundamental characteristics of a translational scientist. ACS Pharmacol Transl Sci. 2019;14(3):213–216. doi: 10.1021/acsptsci.9b00022. PMC7088880 32259057

