# Supplementary material for: Evaluating translational science knowledge gains following an online short course for a general scientific audience
Source: J Clin Transl Sci. 2025 Jan 21;9(1):e16. doi: 10.1017/cts.2024.585 (PMC11795860; doi:10.1017/cts.2024.585)
Supplement: Vogel et al. supplementary material [file S2059866124005855sup001.docx]

**Supplementary Materials for, “Evaluating Translational Science Knowledge Gains following an Online Short Course for a General Scientific Audience**

This document includes the baseline survey instrument and endpoint survey instrument referenced in the manuscript.

**BASELINE SURVEY INSTRUMENT**

| **Q#** | | **Survey Question** | |
| --- | --- | --- | --- |
| **1** | | **Do you wish to participate in the survey?** (required question)  Yes, I consent to participate (skip pattern: go to question 2)  No, I decline to participate (skip pattern: go to closing screen) | |
| **Please answer the following questions about your participation in this course.** | | | |
| **2** | | **How did you hear about this course?** (e.g., FAES catalogue, particular listserv or website) ________________________________________________________________ | |
| **3** | | **Which of the following best reflects your learning goals for your participation in this course? (Select all that apply)**  Get an introduction to translational science  Get an introduction to drug discovery and development  Obtain knowledge and skills that I can apply in my current work  Obtain knowledge and skills that I can apply in my future work  Learn how others are teaching translational research skills, to help me develop/enhance a course on this topic  Other: _______________________________________________________________________ | |
| **We are interested in learning about course participants’ background related to the course content. Please answer the following related questions.** | | | |
| **4** | | **How much translational research experience have you had, to date?** (required question)  Less than 1 year  1 -2 years  3 -5 years  6-10 years  More than ten years | |
| **5** | | **Does your current work contribute to translational research? (e.g., laboratory, clinical, population-level, administrative, or training activities related to translational research)** (required question)  Yes  No  N/A – I’m a student | |
| **6** | | **Do you currently teach a course in skills for translational research?** (required question)  Yes (go to question 7)  No (go to question 8) | |
| **7** | | (Skip pattern: If answered “yes” to prior question, ask this) **What is the focus of this course?**  **________________________________________________** | |
| **8** | | **Please check all that apply, related to your background relevant to cancer biology and cancer care.** (required question)  I have academic training in cancer biology  I have been involved in conducting cancer biology research  I have been involved in conducting other cancer research  I have been involved in providing cancer patient care  None of the above | |
| **9** | | **Please check all that apply, related to your background relevant to drug discovery and development.** (required question)  I have academic training in drug discovery and development  I have been involved in conducting drug discovery and development research  I have been involved in business, administrative, or legal work around drug discovery or development  None of the above | |
| **We are interested in learning more about course participants’ pre-course understanding of Translational Science topics that will be covered in the course. Please help us by answering the following questions.**  **The same questions will be asked at the end of the course, to assess how effective this course was in teaching this material. Your candid responses will help us improve the course for future participants.** | | | |
| **10** | **Please rate your current knowledge of each of the following topics in translational science. (Five response options: No Knowledge, Slight Knowledge, Moderate Knowledge, Significant Knowledge, Expert Knowledge)** | | |
|  | ***GENERAL CONCEPTS IN TRANSLATIONAL SCIENCE*** | | |
|  | 1. The stages of research on the translational spectrum, and how they interact | | |
|  | 1. The difference between “translational research” and “translational science” | | |
|  | 1. The definition of “**scientific** principles of translational science” | | |
|  | 1. The definition of “**operational** principles of translational science” | | |
|  | 1. Key **scientific** challenges in translational research that translational science aims to address | | |
|  | 1. Key **operational** challenges in translational research that translational science aims to address | | |
|  | ***TRANSLATIONAL SCIENCE APPROACHES, STRATEGIES AND METHODS*** | | |
|  | 1. Approaches to advance research in **under-investigated areas of science** (e.g., where there are research gaps, disincentives) | | |
|  | 1. Approaches to develop research initiatives that **address unmet patient or population health needs** (e.g., rare diseases, health disparities) | | |
|  | 1. Strategies to conduct research that **produce solutions to challenges that have stymied multiple projects** (e.g., roadblocks, bottlenecks, disincentives) | | |
|  | 1. Strategies to increase **creativity and innovation** in translational research | | |
|  | 1. Approaches to **increase the** **efficiency or accelerate the pace** of translational research | | |
|  | 1. Approaches to take **evidence-informed risks** in scientific goals, research questions, and methods, toward advancing translational research | | |
|  | 1. Methods to **engage in cross-disciplinary science** that integrates disciplinary expertise to produce research that is more innovative, impactful, and/or accelerates advancement along the translational spectrum | | |
|  | 1. Approaches to **compose effective cross-disciplinary and cross-agency science teams** to advance translational research projects | | |
|  | 1. Strategies for **enhancing collaborative** **interactions** among members of translational research teams in ways that help to increase the team’s scientific effectiveness | | |
|  | 1. Approaches to form and sustain **effective and productive cross-agency partnerships (among government agencies, universities, and/or industry)** to advance translational research | | |
|  | 1. Approaches to develop and sustain **a culture of science** at the level of an initiative, organization, or agency that enables scientists to engage in the strategies and approaches listed in this table, toward advancing translational research. | | |
| **We are interested in learning more about course participants’ pre-course understanding of Scientific Topics that will be covered in the course. Please help us by answering the following questions.**  **The same questions will be asked at the end of the course, to assess how effective this course was in teaching this material. Your candid responses will help us improve the course for future participants.** | | | |
| **11** | | | **Please rate your current knowledge of each of the following topics in preclinical translational science. (Five response options: No Knowledge, Slight Knowledge, Moderate Knowledge, Significant Knowledge, Expert Knowledge)** |
|  |  |  | ***DRUG DISCOVERY APPROACHES*** |
|  |  |  | That there are *a priori* criteria that can be used to identify promising translational research projects that are more likely to lead to success in drug discovery and development |
|  |  |  | That there are different drug discovery approaches (e.g., phenotypic and target-based) that have different advantages and disadvantages |
|  |  |  | The scientific value of high-throughput screening to catalyze new directions for existing research programs |
|  |  |  | The use of multiple, rigorous target identification methods following phenotypic drug discovery approaches increases confidence that the predominant mechanism of action has been identified |
|  |  |  | That target identification following phenotypic drug discovery can generate new avenues for research |
|  |  |  | The critical importance of using multiple approaches to validate findings across all stages of the preclinical research project (e.g., high-throughput screening, target identification, toxicology testing) |
|  |  |  | ***DRUG DEVELOPMENT APPROACHES*** |
|  |  |  | The fact that medicinal chemistry approaches can be optimized to create compounds that can be used both to test a biological hypothesis and to advance the research from drug discovery to drug development |
|  |  |  | That there are limitations of individual *in vitro* and *in vivo* models when attempting to recapitulate the complexity of human disease, which can be addressed by examining data in aggregate across multiple models |
|  |  |  | That while pharmacology and toxicology testing must produce particular data to fulfill requirements for the Investigational New Drug Application to the FDA, there is nonetheless the opportunity to introduce new approaches that increase predictive accuracy and create efficiencies in how data are produced |
|  |  |  | ***CLINICAL TRIALS*** |
|  |  |  | There are principles of good clinical practice that guide the design and implementation of clinical trials |
|  |  |  | That clinical trial design and implementation must comply with guidelines from both federal and institutional oversight and regulatory bodies |
|  |  |  | ***COLLABORATIONS AND PARTNERSHIPS*** |
|  |  |  | That drug discovery and development are two different phases of a project requiring involvement of scientists with different scientific expertise. |
|  |  |  | There are approaches for team-based science, including approaches to setting goals, communication, and coordination of activities, that help to increase team effectiveness |
|  |  |  | There are particular approaches for implementing collaboration agreements and patent strategies that help to facilitate effective multi-agency partnerships in translational science |
| **Please complete the following brief questions, so we can learn more about our course participants.** | | | |
| **12** | | | **Which of the following best describes your work sector?** (required question)  Academia (go to question 15)  Government (go to question 13)  Industry/business/private sector (go to question 15)  Non-profit/non-governmental organization (go to question 15)  None; I am retired (go to question 15)  Other: ______________________________________ |
| **13** | | | (Skip pattern: If answered “Government” to prior question, ask this) **Do you currently work at the National Institutes of Health? (Please select “yes” whether you are a federal employee, contractor, or fellow)**  Yes (go to question 14)  No (go to question 15) |
| **14** | | | (Skip pattern: If answered “Yes” to NIH question, ask this) **At which NIH Institute or Center do you work?**  CC  CIT  CSR  FIC  NCATS  NCCIH  NCI  NCMRR  NEI  NHGRI  NHLBI  NIA  NIAAA  NIAID  NIAMS  NIBIB  NICHD  NIDA  NIDCD  NIDCR  NIDDK  NIEHS  NIGMS  NIMH  NIMHD  NINR  NINDS  NLM  OD, NIH |
| **15** | | | **What is the highest degree you have received to date?** (required question)  Bachelors  Masters  MD  PhD  MD/PhD  Other: please specify your highest degree here ___________________ |
| **16** | | | **In what discipline is your highest degree?** (required question)  Biology  Biochemistry  Chemistry  Computer Science or Informatics  Medicine  Pharmacology or Toxicology  Other: please specify the discipline of your highest degree here ___________________ |
| **17** | | | **How many years has it been since you received your highest degree?**  0-5 years  6-10 years  11-15 years  16-20 years  More than 20 |
| **18** | | | **Are you currently a fellow?**  Yes (go to question 19)  No (go to question 20) |
| **19** | | | *(Skip pattern: If answered “yes” to prior question, ask this)* **Which of the following best describes your fellowship?** (required question)  Post-baccalaureate Fellowship  Pre-doctoral Fellowship (currently in a doctoral training program)  Post-doctoral Fellowship (recently completed doctoral program)  Research Fellowship (fellowship that follows a post-doc, or mid-career fellowship)  Other: please specify your fellowship type here: ___________________ |
| **20** | | | **Are you currently enrolled as a student at a degree-granting institution (i.e., a university or college)?**  Yes (go to question 21)  No (go to question 23) |
| **21** | | | (Skip pattern: If answered “yes” to Student yes/no question, ask this) **For what degree are you currently studying?** (required question)  Bachelors  Masters  PhD  MD  MD/PhD  Other: please specify the degree program here ____________________________________________ |
| **22** | | | (Skip pattern: If answered “yes” to Student yes/no question, ask this) **In what discipline is your current degree program?** (required question)  Biology  Biochemistry  Chemistry  Computer Science or Informatics  Medicine  Pharmacology or Toxicology  Other: please specify the discipline of your highest degree here ___________________ |
| **23** | | | **Are you currently studying or working in the US or abroad?**  United States  Abroad  If abroad, enter country name here: ________ |
| **24** | | | **If you are a US resident or citizen -- what is your racial/ethnic identification? Please select all that apply.**   - American Indian or Alaska Native - Asian - Black or African American - Hispanic or Latino - Native Hawaiian or Other Pacific Islander - White - Other (please state here) - Prefer not to say - Does not apply --- I am not a US resident or citizen |
| **25** | | | **What is your gender identity?**   - Man - Woman - Nonbinary - Transgender - Other (please state here) - Prefer not to say |
|  | | | **Closing Screen: Thank you very much for your time.** |

**ENDPOINT SURVEY INSTRUMENT**

| **Q#** | **Survey Question** |
| --- | --- |
| **1** | **Do you wish to participate in the survey?** (required question)  Yes, I consent to participate (skip pattern: go to question 2)  No, I decline to participate (skip pattern: go to closing screen) |
| **Please answer the following questions about your participation in the course.** | |
| **2** | **How many of the course lectures did you listen to?**  Response options will be on a five-point Likert scale: None (0%), Few (1 - 24%), Some (25 - 49%), Many (50 - 74%), Most to All (75 - 100%) |
| **3** | **How many of the required readings did you complete?**  Response options will be on a five-point Likert scale: None (0%), Few (1 - 24%), Some (25 - 49%), Many (50 - 74%), Most to All (75 - 100%) |
| **4** | **How many of the recommended readings did you complete?**  Response options will be on a five-point Likert scale: None (0%), Few (1 - 24%), Some (25 - 49%), Many (50 - 74%), Most to All (75 - 100%) |
| **5** | **How many of the discussion board assignments did you participate in?**  Response options will be on a five-point Likert scale: None (0%), Few (1 - 24%), Some (25 - 49%), Many (50 - 74%), Most to All (75 - 100%) |
| **6** | **How often did you post a “Live Q and A session” question related to that week’s course content?**  Response options will be on a five-point Likert scale: None of the time (0%), A few times (1 - 24%), Some of the time (25 - 49%), Many times (50 - 74%), Most to All of the time (75 - 100%) |
| **7** | **Which of the “Live Q and A sessions" with course speakers did you listen to? Please include both "live" and recorded Q and A sessions you listened to.** (required question)  None  Week 4  Week 7  Both week 4 and week 7 |
| **8** | **How many times did you discuss the concepts or content that you learned in the course outside of class at least once/week? (e.g., with colleagues or friends)**  Response options will be on a five-point Likert scale: None of the time, A few times (1 – 2 weeks), Some of the time (3 weeks), Many times (4-5 weeks), Most to All of the time (6-7 weeks) |
| **Please answer the following questions about the design of the course.** | |
| **9** | **How effective was the online format to teach the content?** (required question)  Response options will be on a four-point Likert scale: Not at all effective, somewhat effective, moderately effective, very effective |
| **10** | **How effective was the case study approach to teach the course content?** (required question)  Response options will be on a four-point Likert scale: Not at all effective, somewhat effective, moderately effective, very effective |
| **We are interested in learning more about course participants’ post-course understanding of Translational Science topics that were covered in the course. Please help us by answering the following questions.**  **The same questions were asked at the start of the course. We are asking them again here, to assess how effective this course was in teaching this material. Your candid responses will help us improve the course for future participants.** | |
| **11** | **Please rate your current knowledge of each of the following topics in translational science. (Five response options: No Knowledge, Slight Knowledge, Moderate Knowledge, Significant Knowledge, Expert Knowledge)** |
|  | ***GENERAL CONCEPTS IN TRANSLATIONAL SCIENCE*** |
|  | The stages of research on the translational spectrum, and how they interact |
|  | The difference between “translational research” and “translational science” |
|  | The definition of “**scientific** principles of translational science” |
|  | The definition of “**operational** principles of translational science” |
|  | Key **scientific** challenges in translational research that translational science aims to address |
|  | Key **operational** challenges in translational research that translational science aims to address |
|  | ***TRANSLATIONAL SCIENCE APPROACHES, STRATEGIES AND METHODS*** |
|  | Approaches to advance research in **under-investigated areas of science** (e.g., where there are research gaps, disincentives) |
|  | Approaches to develop research initiatives that **address unmet patient or population health needs** (e.g., rare diseases, health disparities) |
|  | Strategies to conduct research that **produce solutions to challenges that have stymied multiple projects** (e.g., roadblocks, bottlenecks, disincentives) |
|  | Strategies to increase **creativity and innovation** in translational research |
|  | Approaches to **increase the** **efficiency or accelerate the pace** of translational research |
|  | Approaches to take **evidence-informed risks** in scientific goals, research questions, and methods, toward advancing translational research |
|  | Approaches to **engage in cross-disciplinary science** that integrates disciplinary expertise to produce research that is more innovative, impactful, and/or accelerates advancement along the translational spectrum |
|  | Approaches to **compose effective cross-disciplinary and cross-agency science teams** to advance translational research projects |
|  | Strategies for **enhancing collaborative** **interactions** among members of translational research teams in ways that help to increase the team’s scientific effectiveness |
|  | Approaches to form and sustain **effective and productive cross-agency partnerships (among government agencies, universities, and/or industry)** to advance translational research |
|  | Approaches to develop and sustain **a culture of science** at the level of an initiative, organization, or agency that enables scientists to engage in the strategies and approaches listed in this table, toward advancing translational research |
| **We are interested in learning more about course participants’ post-course understanding of Translational Science topics that were covered in the course. Please help us by answering the following questions.**  **The same questions were asked at the start of the course. We are asking them again here, to assess how effective this course was in teaching this material. Your candid responses will help us improve the course for future participants.** | |
| **12** | **Please rate your current knowledge of each of the following topics in preclinical translational science. (Five response options: No Knowledge, Slight Knowledge, Moderate Knowledge, Significant Knowledge, Expert Knowledge)** |
|  | ***DRUG DISCOVERY APPROACHES*** |
|  | That there are *a priori* criteria that can be used to identify promising translational research projects that are more likely to lead to success in drug discovery and development |
|  | That there are different drug discovery approaches (e.g., phenotypic and target-based) that have different advantages and disadvantages |
|  | The scientific value of high-throughput screening to catalyze new directions for existing research programs |
|  | The use of multiple, rigorous target identification methods following phenotypic drug discovery approaches increases confidence that the predominant mechanism of action has been identified |
|  | That target identification following phenotypic drug discovery can generate new avenues for research |
|  | The critical importance of using multiple approaches to validate findings across all stages of the preclinical research project (e.g., high-throughput screening, target identification, toxicology testing) |
|  | ***DRUG DEVELOPMENT APPROACHES*** |
|  | The fact that medicinal chemistry approaches can be optimized to create compounds that can be used both to test a biological hypothesis and to advance the research from drug discovery to drug development |
|  | That there are limitations of individual *in vitro* and *in vivo* models when attempting to recapitulate the complexity of human disease, which can be addressed by examining data in aggregate across multiple models |
|  | That while pharmacology and toxicology testing must produce particular data to fulfill requirements for the Investigational New Drug Application to the FDA, there is nonetheless the opportunity to introduce new approaches that increase predictive accuracy and create efficiencies in how data are produced |
|  | ***CLINICAL TRIALS*** |
|  | There are principles of good clinical practice that guide the design and implementation of clinical trials |
|  | That clinical trial design and implementation must comply with guidelines from both federal and institutional oversight and regulatory bodies |
|  | ***COLLABORATIONS AND PARTNERSHIPS*** |
|  | That drug discovery and development are two different phases of a project requiring involvement of scientists with different scientific expertise. |
|  | That there are particular approaches for team-based science, including approaches to setting goals, communication, and coordination of activities, that help to increase team effectiveness |
|  | That there are particular approaches for implementing collaboration agreements and patent strategies that help to facilitate effective multi-agency partnerships in translational science |
|  |  |
|  | **Please complete the following questions asking for your perspectives on the course.** |
| **13** | **How much did participating in this course influence the scientific or operational approaches you intend to use in your future work?** (required question)  Response options will be on a four-point Likert scale: Not at all, Slightly, Moderately, A great deal |
| **14** | **Please elaborate on your answer here. If applicable, please tell us about the influence of the course on the scientific or operational approaches you intend to use in your future work.** (Optional question - open ended text response) |
| **15** | **How much did participating in this course influence your goals for your future work?** **Examples might include, but are not limited to the kinds of research activities you would like to engage in or your broader scientific or professional goals.** (required question)  Response options will be on a four-point Likert scale: Not at all, Slightly, Moderately, A great deal |
| **16** | **Please elaborate on your answer here. If applicable, please tell us about the influence of the course on your goals for your future work.** (Optional question - open ended text response) |
| **17** | **We hope to provide course participants with a unique window into the translational science process. Please rate the degree to which this course achieved this aim.** (required question)  Response options will be on a four-point Likert scale: Not at all, Slightly, Moderately, Completely |
| **18** | **Please rate the value of this course to you, overall.** (required question)  Response options will be on a four-point Likert scale: Not at all valuable, Slightly valuable, Moderately valuable, Extremely valuable |
| **19** | **Please use this space to share your thoughts on why you rated the course this way.** (optional question - open ended text response.) |
| **20** | **Course participants are an ideal source of ideas for enhancing the course. Please use this space to provide your recommendations. Feel free to comment on any aspect of the course. (Examples include but are not limited to: content/topics covered, design of the course, speakers, delivery, format.)** (optional question - open ended text response.) |
| **21** | **If we were to expand this course to a 2-credit offering, we would add more lectures. What current content should we elaborate on? What additional topics should we cover, that are not covered already?** |
| **22** | **To help us learn more, would you be interested in elaborating on your responses in a brief interview with an NCATS Education Branch staff member?** (required question.)  Yes (go to question 22)  No (go to closing screen) |
| **23** | **Please use this space to provide your name and email address. You will be contacted by a member of the NCATS Education Branch. Please Note: if you provide your name here, it will be separated from the rest of your survey responses, which are anonymized**. (optional question.) |
| **24** | **Closing Screen: Thank you very much for your time.** |
